# Redefining standards—response to: introductions of technological innovations in neurosurgery

**DOI:** 10.1007/s00701-021-04853-9

**Published:** 2021-05-04

**Authors:** Anna L. Roethe, Thomas Picht

**Affiliations:** 1 Image Guidance Lab, Department of Neurosurgery, Charité - Universitätsmedizin Berlin, Charitéplatz 1, 10117 Berlin, Germany; 2 Cluster of Excellence Matters of Activity. Image Space Material, Humboldt-Universität Zu Berlin, Berlin, Germany; 3 Berlin Simulation and Training Center (BeST), Charité-Universitätsmedizin Berlin, Berlin, Germany

We would briefly like to seize the opportunity and comment on Mr. Ammirati’s recent editorial on technological innovations in neurosurgery [1] as we consider the ongoing discussion behind the topic consistently relevant.

Back when we decided to compare a hybrid microscope comprising an exoscope mode with a conventional microscope by the same manufacturer in a clinical study, the leading question was how to assess the multiple dimensions of the operative device beyond previously reported qualities attributed to exoscopes in general [13]. We also intended to perform a comparison of both an evaluation under laboratory conditions and under the conditions of clinical routine. Furthermore, the chosen hybrid microscope allowed for direct comparison of two different visualization modes – one novel, the other established standard – in a single device. As prior preclinical and clinical investigations mapped out, the most obvious advantages of exoscopic surgery include improved ergonomics and sharing of intraoperative information for improved communication and teaching [2, 3, 14]. Our intent was to analyze the visual and haptic quality as well as the potential restrictions to established operative practice. Ultimately, our data showed a varying impact on intraoperative visualization and task performance. Aside from safety and general feasibility, we were able to present some minimal requirements for optimal use (setup, distance, eligible cases). This included the necessity of adequate user training as demonstrated by the results of the system usability scale (SUS). The performance and dexterity of the one expert user who did the most exoscopic surgeries in the study was equal in speed and motor coordination as rated by a blinded expert based on microscope recordings.

Despite a broad interest in the technology in the recent decade, to date, no exoscopic device has fully proven superiority to the surgical microscope [8, 11, 12]. When a novel device is being introduced to the market, clinical trials usually report on safety, feasibility, and comparability to an established standard. The subsequent question, however, has to be: how much better should a new standard be in order to qualify as a standard? Should it enable surgical practices heretofore unfeasible? Does it have to come with a significantly reduced surgical morbidity and mortality? Is an associated reduction of surgical time and/or cost indispensable? Do long-term effects of improved posture on physical well-being justify the introduction of a new standard?

With the call for introducing randomized trials in technology innovation and the development of the IDEAL framework [6, 7, 9, 10], the ground for future research in technological innovation has been prepared. Many more discussions need to follow. Ammirati pointed out how sharing negative results is important for public discourse on technology innovation. We entirely agree with this; this is why our framework for technology evaluation, like many others, is being developed in a multidisciplinary research lab and continuously adjusted and expanded in other contexts and devices. A critical approach allows for affirmation of features that proved to work while reporting on restrictions or drawbacks; just like the development process, it is iterative by definition [4–6]. Hence, reviewing the armamentarium of methods is only the first important step. Present and future collaboration models with industry partners need to be under review as well. Therein lies an opportunity for all stakeholders to redefine overcome roles in the innovation process. We further agree with Ammirati that using the opinion of medical experts as the sole argument for the introduction of new medical technology is an outdated concept. In this context, it is long overdue to restructure clinical contract research, not as a paid endorsement of industry developments but as an indispensable, independent, and iterative benchmarking during the whole process of technological innovation. Technological change in operative neurosurgery and its investigation by users remains a heterogenous and ambitious project. It closely involves several participants aside from a surgeon and a manufacturer. As neurosurgeons, working with, towards and sometimes against technological innovation means a constant refinement of methods in exchange with our industry partners and research community.
